# Barriers to family involvement in the care of patients with chronic mental illnesses: A qualitative study

**DOI:** 10.3389/fpsyt.2022.995863

**Published:** 2022-10-19

**Authors:** Raziye Dehbozorgi, Malek Fereidooni-Moghadam, Mohsen Shahriari, Ebrahim Moghimi-Sarani

**Affiliations:** ^1^School of Nursing and Midwifery, Isfahan University of Medical Sciences, Isfahan, Iran; ^2^Research Center for Psychiatry and Behavior Science, Shiraz University of Medical Sciences, Shiraz, Iran

**Keywords:** family involvement, barriers, mental illnesses, qualitative study, patients

## Abstract

**Introduction:**

Caregivers are patients' family members or intimate friends who take care of individuals suffering from chronic mental illnesses without being paid. Evidence has supported the role of family-centered collaborative care in the treatment of patients with chronic mental illnesses. It has also been emphasized by national policies. However, carrying out this type of care is accompanied by challenges in Iran. Considering the importance of family participation in taking care of these patients as well as the necessity to determine its effective factors, the present study aimed to assess the barriers to family involvement in the care of patients with chronic mental illnesses.

**Method:**

A conventional content analysis was used to conduct this qualitative study. Thirty four health care providers, patients, and caregivers were interviewed unstructured in-depth face-to-face using purposive sampling. Until saturation of data, sampling and data analysis were conducted simultaneously. Graneheim and Lundman's method was used to record, transcribe, and analyze the interviews.

**Result:**

The results showed that there were many barriers to the collaboration of family in the care of patients with chronic mental illnesses. Accordingly, four main categories and twelve subcategories were extracted from the data as follows: “family-related barriers”, “treatment-related factors”, “disease nature threatening care”, and “mental disease-associated stigma in the society”.

**Conclusion:**

The findings presented the barriers to family centers' collaborative care in patients with chronic mental illnesses and the necessary components of family involvement in the care to be used by healthcare managers and policymakers. The reported barriers emphasize the need for the development of structured approaches whose implementation is easy for health care providers, does not require a lot of time and resources, and can improve patient and family outcomes.

## Introduction

Chronic Mental Illnesses (CMIs) such as schizophrenia, bipolar mood disorder (BMD), and major depressive disorder (MDD) have long disease courses as well as cognitive disorders, which can lead to physical and social consequences such as the loss of function and even death (1–3). According to the latest study, Schizophrenia prevalence worldwide increased from 13.1 million cases in 1990 to 20.9 million cases in 2016 (4). In 2017, there were 25,8 million incidents of depression worldwide, an increase of 49.86% from 172 million in 1990 (5). Also, the number of people with BMD worldwide increased from 32.7 million in 1990 to 48.8 million in 2013 (6). Cognitive and functional disabilities impose a great burden on caregivers. Caring for someone with mental illness affects caregivers emotionally, financially, and physically, according to a recent Iranian qualitative study. Caregivers are patients' family members or intimate friends who support and take care of them without being paid (7, 8). Caregivers can help therapists diagnose the initial symptoms of disease recurrence and cooperate in mental health interventions in patients' care programs (9, 10).

Obviously, an effective evidence-based intervention such as participating family in the care requires skills, knowledge, and continuous examination of patients and caregivers to achieve a stable mental health status (11, 12). Family collaboration is one of the main components of family-centered care that considers the family as a specialist and partner in all dimensions of care provision (13).

According to the World Health Organization (WHO), Ministry of Health (MOH), Scottish Intercollegiate Guidelines Network (SIGN), and National Institute for Health and Clinical Excellence (NICE), families have to be involved in pharmacotherapy and psychotherapy interventions for supporting patients suffering from CMIs (11, 14).

Evidence has indicated that caregivers' participation reduces the need for re-hospitalization, enhances the life quality of patients, and decreases the risk of recurrence of severe mental disorders (10, 15). However, this goal has not been achieved in numerous clinical services and caregivers are deprived of participation in the treatment process (16–18). It seems that planning for hospitalized patients creates further challenges for the participation of caregivers. In other words, due to the shortage of information and lack of cooperation in clinical decision-making during the hospital stay, caregivers face problems in supporting their patients after discharge. Therefore in order to achieve the effective involvement of caregivers in care provision, cooperation is required on the part of patients, caregivers, and Health Care Professionals (HCPs) (19, 20).

Up to now, the studies conducted on caregivers' cooperation have mainly focused on clinical ideas and models guided by therapists (21, 22). Nonetheless, few studies have been done on the opinions of patients, families, and HCPs (23, 24). Therefore, the present study aims to explore the challenges and barriers of family involvement in the care (FIC) in hospital environments which means the participation and involvement of patient caregivers in the process of diagnosis to treatment and rehabilitation of patients with chronic mental disorders (24).

## Methods

### Aim

The present study aimed to determine the barriers to family involvement in the care of patients with chronic mental illnesses.

### Study design

This study used the qualitative content analysis approach. Essentially, content analysis is the process of perception, interpreting, and conceptualizing qualitative data (25).

### Participants

Out of the 34 interviews, 8 were performed with patients, 11 with caregivers, and 15 with HCPs. The participants' demographic characteristics have been presented in Table 1.

**Table 1:** The participants' socio-demographic characteristics.

**Gender**	**Female *n* (%)**	**Male *n* (%)**
Patients	3 (37.5%)	5 (62.5%)
Caregivers	9 (81.81%)	2 (18.18%)
HCPs	11 (73.33%)	4 (26.66%)
Age	Mean (standard deviation)	
Patients	30.5 (5.049)	
Caregivers	44.45 (10.73)	
HCPs	41.93 (5.85)	
Caregiver's relation with the patient	Mean *n* (%)	
Mother	4 (36.36%)	
Father	1 (9.09%)	
Wife	1 (9.09%)	
Sister	2 (18.18%)	
Daughter	2 (18.18%)	
Brother	1 (9.09%)	
Diagnosis	Mean *n* (%)	
Schizophrenia	4 (50%)	
Major depressive disorder	1 (12.5%)	
Bipolar mood disorder	3 (37.5%)	
Experience of working in the field of acute mental healthcare (years)	Mean (standard deviation)	
HCPs	11.93 (4.49)	

Participants for the study included: (a) patients with CMIs including schizophrenia and BMD, (b) family caregivers include family members and friends, and (c) HCPs include psychiatrists, psychologists, psychiatric nurses, social workers, and occupational therapists. The participants were selected through purposive sampling. The participants who met the inclusion criteria and were willing to take part in the research were provided with the details of the study including study objectives as part of the informed consent process before getting their written informed consent.

The inclusion criteria of the study for the patients were being able to speak Persian, having a history of hospitalization due to CMIs during the past 5 years or referral to a clinic for follow-up, having at least one family caregiver, age above 18 years, is conscious, not being in the acute phase of the disease, being able to communicate, not having cognitive or speech problems, delusion, and hallucination, and being able to complete the written informed consent form. The inclusion criteria for the caregivers were having experience in caring for individuals hospitalized due to CMIs (during the past 5 years), possessing the necessary communication skills, not having cognitive, speech, or mental problems, being above 18 years of age, and being willing to participate. Finally, HCPs must have worked with hospitalized psychiatric patients for at least 3 years to qualify to enroll in the research study.

### Data collection

Semi-structured interviews were conducted with 34 participants including patients, caregivers, and HCPs from 30 December 2020 to 25 August 2021. The interviews were individually conducted until data saturation was achieved. To study the barriers to family involvement in the care of patients with CMIs, 34 semi-structured interviews were conducted in the in-patient and outpatient psychiatric educational settings affiliated with Isfahan University of medical sciences. In addition, direct observations were done and field notes were completed. In order to identify domains, we developed two to four open-ended questions for each of the three groups (Box 1). The interviews lasted for 30–70 min. During the interviews with the patients, the main question was related to their expectations from HCPs and caregivers regarding care services, needs, and problems as well as their barriers. While interviewing the HCPs, their perspectives concerning patients' and caregivers' needs were explored and their experiences of teamwork were evaluated. Finally, the caregivers were asked about their expectations and needs for cooperation in care as well as the existing problems and obstacles in this field. The interviews were recorded using a voice recorder and were transcribed immediately.

Box 1 **Open-ended questions tailored to participants' groups (patients, informal caregivers and Patients' open-ended questions.**

Patients.

- “Please talk about your expectations of the treatment team and family about your care and the problems you feel about it.”
- “Please describe times when you think it is difficult to take proper care of yourself.”

Informal caregivers' open-ended questions.

- “Please talk about the expectations you have for your involvement in caring for your patient and the problems you feel in doing so.”
- “ Describe actions that you think have threatened, harmed, or violated your involvement in caring for your patient.”

HCP's open-ended questions.

- “Please, if possible, explain how you use the opinion of other members of the treatment team or the patient and his family in developing a care plan?”
- “How do you participate families in the care?”
- “How is this collaboration maintained or ignored?”
- “Please talk about the need for involvement of family in the care.”) The continuation of the interview will be based on participatory answers and exploratory questions.

### Data analysis

Then, the data were analyzed using conventional content analysis. At first, the researcher listened to the interviews several times in order to gain an overall understanding of the participants' statements. After transcription, the texts were coded by one of the researchers. The interview was considered a unit of analysis. In addition, a paragraph, a sentence, or even a word was considered a meaning unit. Based on latent meanings, the meaning units were coded. Then, the codes were compared and classified into more abstract categories on the basis of their similarities and differences. After all, the latent contents of the data were discovered themes were developed. MAXQDA software (2018) was used for coding and organizing the data (25).

### Trustworthiness

The trustworthiness of the data was assessed using the criteria proposed by Lincoln and Guba (26). Accordingly, data credibility was ensured by selecting the participants with maximum variation in terms of age, sex, and experience as well as using multi-level criteria such as interviews with participants at different levels of education, age, and work experience. Prolonged engagement in the field (7 months and 22 days), constant observation (full attention and focus on the observed activities), and triangulation data gathering (interview, observation, and field notes) were also employed to guarantee the credibility of the data.

### Ethical considerations

Isfahan University of Medical Sciences Ethics Committee approved the project (IR.Mui.Research.REC.1399.502) and acquired the necessary licenses. The participants' written and oral informed consent was also obtained before data collection. As well, the interviews were conducted in a way that respected the privacy and comfort of participants. In addition, to ensure the anonymity and confidentiality of the participants' information, a code was given to each of them. In addition, they were assured that their treatment would not be negatively affected if they withdraw from the study. All words from the transcripts were transcribed, and the codes extracted from the participants were exact. The study approval and permission to conduct the study by the Ethics Committee, School of Nursing and Midwifery as well as from those of the health centers and unit managers were sought prior to the data collection.

## Results

Four main categories with subcategories were extracted from the analysis of data that included family-related barriers, treatment-related factors, disease nature threatening care, and mental disease-associated stigma in society (Table 2).

**Table 2:** Summary of study findings.

**Theme**	**Category**	**Subcategory**
Barriers of family-centered collaborative care	Family-related barriers	Family's insufficient insight into mental disorders
		Family's negligence of appropriate care
		Familial crises and conflicts
		Patient rejection
		Preparatory challenges of care
	Treatment-related factors	Factors related to structure
		Factors related to process
		Factors related to outcomes
	Disorder related factors	Variety of patient symptoms as a barrier
		Lack of acceptance of the disease and the treatment
	Stigma associated with mental disorders in the society	Reduced family participation in care following stigmatization
		Mental disease-associated taboo as an obstacle against macro level supports
		Hiding the disease due to the related stigma

### Family-related barriers

Family-related barriers were found to be among the most important challenges in the application of family involvement in the care of patients with CMIs. This category consisted of five subcategories, namely the family's insufficient insight into mental disorders, family's care challenges, family crises and conflicts, and patient rejection (Table 2).

#### Family's insufficient insight into mental disorders

Inadequate awareness of the symptoms, treatment, and role of caregiving by families posed a barrier to family-centered participatory care. The patient's daughter with MDD, who also has panic disorder, said:

“*Families don't understand mental illness, even my wife, who has been a witness to my illness for 12 years, sometimes she can't understand, she says maybe you are suggesting, maybe you can focus on yourself and help, but these attacks come.”* (C11P34I1) (Participants are coded as “C” for caregivers, “P” for participants).

Another caregiver stated:

“*My husband was hospitalized last year and had to receive a shock. However, his brother discharged him without informing us. We became really upset; we said that the treatment course was not completed and he was not well. When he came home, he wasn't well, he even got worse”* (C6P9I1).

#### Family's negligence of appropriate care

Some HCPs pointed to caregivers' negligence in patient care, and some pointed to caregivers' excessive involvement in their patient care. A family's insufficient support for the patient, lack of follow-up, insufficient presence by the patient, and excessive but ineffective cooperation, as well as frequently changing the physician, are all examples of family neglect.

“*Some patients say that they would tolerate cancer more easily than bipolar mood disorder or schizophrenia because it seemed that even their families did not accept them”* (Participants are coded as “PS” for psychiatrists) (PS1P27I1).

One of the participants expresses the family's negligence in caring for this

“*During our home visits, we realized that the patients who were hospitalized frequently consumed their medications without following the orders. For example, a patient had taken the medications one day, but not on the next day. Another patient was hospitalized several times, but the family did not have complete information about the disease and the medicines”* (PS1P27I1).

Participant No.3 points to the challenge of excessive but ineffective cooperation of caregivers.

“*Before the COVID-19 pandemic, the families could be divided into three groups. Some families could not be separated from their patients. They were so dependent on their patients that they disrupted the treatment process”* (Participants are coded as “N” for nurses) (N2P3I1).

#### Family crises and conflicts

According to the participants, in some cases, the existence of serious mental disorders in other family members, divorce in the family, legal problems in the family, death of a family member, family's convulsive atmosphere, parents' excessive strictness, verbal or physical punishment on the part of parents, problems in relationships among family members, unsympathetic family members, and marital conflicts, act as family-related barriers affecting their cooperation in the care of their family members:

One of the patients who suffered from MDD believed that having an accident leading to a person's death was the reason for the increased consumption of Zolpidem and the worsening of the disease:

“*Two and a half years ago, I had a terrible accident that resulted in another person's death. It was very difficult for me. I went to jail, but that was not important; the scene mattered to me and I started moping. It was highly effective in my consumption because when I took the medication, I felt carefree”* (Participants are coded as “P” patients) (P1P15I1).

Another patient with schizophrenia was not satisfied with his/her caregiver's behaviors:

“*I feel that my stepmother's behavior is humiliating. She humiliates me with her words and I have become weaker and weaker over time”* (P8P33I1).

#### Patient rejection

Patient rejection was another important category of the family-related barriers to collaborative care, which involved a lack of a supportive caregiver, family's mental disorders, patient's chronic mental disorders, and lack of insight into patient rejection.

Considering the lack of support for these patients, an experienced psychologist maintained:

“*they are the most oppressed patients in our society; they are even rejected by their families. We had a family with high financial status, but they left their patient because he had schizophrenia. The woman said that I was the only one for whom she waited because I was the only one who had not left them. No one asked them how they were, not their brothers, not their sisters”* (PS1P27I1).

#### Preparatory challenges of care

family members living long distances, a shortage of the required facilities in the hospital, Lack of support for organizations from the family, family's low socioeconomic status, family exhaustion, and poverty, and family's worry about the process of treatment, which was huge obstacles to family involvement in the care.

One of the caregivers referred to living at a long distance as a barrier:

“*When we feel that our patient is doing unreasonable things, we have to start the car and drive for 500 kilometers to reach XXX and ask what we have to do in this situation”* (C7P8I1).

Considering the restlessness and fatigue associated with care, one of the HCPs said:

“*since their patient has a chronic disease and they see the patient in the same situation all the time, they have become restless. When we call to train the family, they say that they know everything or they say that the patient does not change and is always the same”* (N7P22I1).

### Treatment-related factors

This category based on the Donabedian model (27) describes the three subcategories factors related to structure, factors related to process, and factors related to outcomes.

#### Factors related to structure

Another barrier to family involvement in the care was factors related to the structure which consisted of management factors and lack of resources. Management factors such as lack of management of follow-up care and support for patients, cumbersome rules of the health system, and the teleological view of the health system. Lack of resources such as limitations related to hospital environment and equipment, a large number of patients, lack of manpower, facing lack of time to care, and insufficient funds allocated to the mental health system. Regarding the lack of management of follow-up care, the patient's daughter with BMD said:

“*When my patient was discharged from the hospital, I also gave my mobile number, but they did not call me and did not follow up on my patient at all.”* (C2P4I1).

Regarding the cumbersome rules of the health system, a nurse with 22 years of experience in a psychiatric hospital said:

“*... The family is desperate. Especially during the corona crisis, when the family brings the patient to the hospital, the patient is left in the hospital. Making it a rule that the family should not go to the hospital at all, the patient can call the family only once a day. I don't think it's the same as political prisons. The family can call the hospital, now they either answer or not, it depends on the doctor, maybe not, and if not, the family has to wait until the next day.”* (N5P20I1).

Teleological view of the health system, like the treatment-oriented system not the promotion-oriented system, the nurse-oriented system, and interference of the duties of the professions. In connection with the treatment-oriented system, not the promotion-oriented system, one of the employees of the health system said:

“*The point of view of the health system is the treatment-oriented point of view. Their opinion is that you have to build beds, even now we have 22 beds and it is exploding, but with these conditions, they still say that you should add more beds. That is, they are looking for the hospital to have more income and admissions. Many times they are not looking for quality”* (N5P20I1).

The head of the women's affairs department with 16 years of experience said the following about the nurse-oriented system:

“*In my opinion, the system that is being implemented now has more workload on the shoulders of the nurse, the nurse has to coordinate with everyone, for example, the other parts say to enter the information and send the patient, if they should interact more, for example, the work is a kind of division. Be careful, not because you are now a nurse, you will be somehow more oppressed.”* (N6P21I1).

A nurse said in connection with the interference of the duties of the professions:

“*Maybe I can say that in some places, even in the positions related to hospital management, the position of the profession has not yet been determined and what their job is. It is much more evident in the departments and the duties of the professions overlap and it can be said that there is no clear boundary.”* (N5P20I1).

Almost all the HCPs complained about a large number of patients and a shortage of human workforce:

“*Most of the time, like now, we have 31 patients and two isolation wards are full. Our bed occupancy rate is 103%, which is higher than the normal percentage”* (N9P24I1).

#### Factors related to process

The restrictions created in the hospital with the emergence of the Corona crisis, Communication barriers with the patient and family, and Challenges of meeting with a doctor for the family are related factors. The wife of a patient with BMD said the following about the restrictions created to meet with their patient:

“*I really want to come and see him, and now he has been hospitalized for 25-26 days, they don't let us at all. In the beginning, we used to come up near the ward, but when I came yesterday, they didn't let us see our patient at all.”*(C6P9I1).

A nurse with 11 years of experience said the following about communication barriers with the patient and family:

“*We and the doctors are obliged to visit the patient in the room, not in the nurse's station where all the patients are, and after all, the privacy of the patient is not protected and he wants to tell the doctor things that he might not like everyone to hear, and some doctors do not comply with this.”*(N2P3I1).

Some families mentioned the challenges they had faced for visiting a physician:

“*The conditions for meeting the doctor were very bad and awful, meaning that all the patients were listening to each other's histories, standing together, the conditions were very ugly. It did not leave my mind for a moment, I could not even talk to my sister's doctor.” (C1P2I)*.

“*There were times we had questions. We had to wait in front of the ward door so that we could talk to the physician in the corridor”* (C5P11I1).

#### Factors related to outcome

Interruption of face-to-face education of the family, interrupting the process of home visits due to the Corona crisis, non-cooperation of health system employees in participation for any reason and, inadequate training and skills of HCPs are these factors. The psychologist in charge of the home visit said about the interruption of face-to-face education of the family:

“*Unfortunately, since the time of Corona, we don't have the face-to-face training program for families, but it used to be once a month, then it was done weekly, and it was very well received, but unfortunately, we don't have this program anymore.”* (PS1P27I1).

The same psychologist said the following about the home visit program:

“*Now that there is a coronavirus and we can't go to the patient's house because we planned for the health of the patient and the staff to visit their families.”* (PS1P27I1).

A nurse said the following about not participating in care due to her job fatigue:

“*There is not so much work here physically, but mentally and emotionally, it is very tiring and one is not in the mood to think about cooperative care of the patient and the family.”* (N4P9I1).

Regarding inadequate training and skills of psychiatric HCPs with 8 years of experience, he said:

“*In my opinion, the team-oriented approach is very weak, one of the reasons is that we have not been trained to work as a team, and they may even make an intervention that will be the opposite of each other's work and then a problem will arise, but we are trying our best now by training Especially because one part of it is a skill and another part is lack of knowledge to be able to form this central team.” (PG1P26I1)*.

### Disorder related factors

This category contained two subcategories, namely patient's variety of symptoms as a barrier and lack of acceptance of the disease and the treatment, which were extracted from the analysis of the participants' viewpoints.

#### Patient's variety of symptoms as a barrier

This subcategory included mood changes, inappropriate sleep, patient exaggerated behaviors, patient declining function, problems in patient-family relationships, pressure in the family due to the patient's behaviors, the patient's resistance against treatment, and patient's lack of insight.

From the participants' viewpoints, patients' mood changes could be considered an obstacle to family involvement in the care:

“*We have some common issues like mood changes in patients with mania. A patient came and told us that he had nothing to do and we involved him/her in the treatment. However, when s/he was visited by the doctor, s/he said that there wasa lot of work to do yesterday, which made him/her tired. Because of such problems, we do not engage patients in care anymore”* (N2P3I1).

A patient with schizophrenia who had studied medicine talked about a functional decline after graduation:

“*Since 2015 when I graduated, I haven't done anything. I was nervous, I was under pressure at home day and night; I was indirectly under pressure. I didn't like to work and have arguments with patients and I didn't want to continue my studies”* (P8P33I1).

#### Lack of acceptance of the disease and the treatment

This category consisted of patients' unwillingness to refer to treatment centers and avoidance of taking medications, which were expressed by some families.

“*We had to tell lies to take him/her to the hospital, we had a lot of challenges. Once s/he realized in the car that we were going to the hospital. S/he escaped as soon as getting off the car”* (C2P4I1).

### Stigma associated with mental disorders in the society

This category contained four main subcategories; i.e., reduced family participation in care following stigmatization, mental disease-associated taboo as an obstacle against macro level supports, and hiding the disease due to the related stigma. These were regarded as barriers to carrying out family involvement in the care.

#### Reduced family participation in care following stigmatization

Many participants noted the decline in family involvement due to the stigma of psychiatric illnesses in the community.

A psychiatrist said about the effect of stigma:

“*The effect of stigma is very strong because even all our treatments can be affected, and because of that stigma, a person or a family may abandon the treatment, and now, unfortunately, many pieces of trainings may not be strong, and this stigma still exists, and it cannot be affected at all. It was ignored and I think it can be said that in some places it can be much more prominent than our role and even the role of the family.”* (PG1P26I1).

#### Mental disease-associated taboo as an obstacle against macro level supports

Numerous HCPs referred to the lack of macro-level support for patients, which they believed was linked with the stigma resulting from mental disorders.

“*I know a person who is the dean of a university. He had told a benefactor who wanted to build a psychiatric hospital whether he wanted to build a madhouse. He had told him in a humiliating way to spend his money on building a hospital for physical disorders”* (N5P20I1).

#### Hiding the disease due to the related stigma

A large number of participants stated that they did not unveil the disease diagnosis due to the stigma associated with mental disorders.

“*When I had just started working, some patients said that their families were not aware of their problem. They were young and they had just learned to talk about their problems and consult with psychologists. Many of them were afraid of others realizing their problem. In our society, people easily talk about a heart attack or cancer, but not about mental disorders”* (PS1P27I1).

## Discussion

The present study aimed to identify the obstacles against family involvement in care among adults suffering from CMIs using the experiences of patients, caregivers, and HCPs. Based on the findings, all the participants indicated family involvement as the main missed component of family involvement in the care. In the same line, Vimala Samson, Kanagaraj et al. mentioned family as a vital component in the treatment process, which was required to be engaged in care programs (28). In addition, Giacco et al. conducted qualitative research and suggested that caregivers had to take part in patient care immediately after admission to facilitate the patients' cooperation and adherence to treatment (24). In the study by Cohen et al. also, the participants mentioned familial factors as the main obstacle against family involvement in the care, with “family's insufficient insight” being its key subcategory (29). In the current investigation, caregivers' involvement in the treatment process was deeply explored and the challenges were identified.

The current study assessed the caregivers' knowledge of patients' status and emphasized their empowerment for active participation in decision-making for collaborative care. Similarly, Clifton (30) pointed to the importance of family-related problems in collaborative care whose elimination could facilitate family involvement in the care (30). Additionally, the family's insufficient insight into mental disorders was found to be the first familial factor hindering collaborative care. The necessity to enhance families' awareness and knowledge regarding mental diseases has also been emphasized in literature (30, 31). Other subcategories of this important category were negligence of appropriate care and patient rejection by one's family. Care negligence and patient rejection can be attributed to a variety of factors. In the research carried out by Feldman et al. (32). The stigma related to mental disorders was found to be as destructive as the disease symptoms and was mentioned as a reason for family conflicts, job discrimination, and social isolation, which were effective in the reduction of families' cooperation in care services.

Another category expressed by some HCPs and caregivers in the current research was the excessive involvement of families in care, which was considered a barrier to family involvement in the care. Similar results were also obtained by Fiona Pharoah et al. (33). Other subcategories of family-related obstacles included crises, conflicts, and care challenges. Evidence has also revealed the necessity of behavior therapy and psychological interventions for solving conflicts and mental problems amongst family members, eventually improving the quality of care (28, 30, 31, 34). In the present study, one of the challenges observed among the caregivers was the shortage of psychosocial interventions including the disruption of home visits due to the COVID-19 pandemic, lack of telemedicine, inattention to lifelong care, lack of follow-up, and lack of phone support, which had led to the families' dissatisfaction. Fabrizio Starace who was worried about chronic patients wrote a letter to an editor in 2020 and emphasized the necessity to continue psychosocial interventions such as home visits based on the new protocols for patients with CMIs (35).

The current study participants mentioned mental disease-associated stigma in society as another barrier against family involvement in the care. Erwing Goffman (1963) 73rd president of the American Sociological Association, in his seminal work on stigma, distinguishes between two different “sympathetic others”: “those who know from their own experience what it is like to have this particular stigma, and the “wise”, “namely persons who are normal but whose special situation has made them intimately privy to the secret life of the stigmatized individual and sympathetic with it, and who find themselves accorded a measure of acceptance, of courtesy membership of the clan”. According to Goffman, there are two types of wise persons: “those working in an environment which caters either to the wants of those with a particular stigma or to actions that society takes regarding these persons”, and “the individual who is related through the social structure to a stigmatized individual—a relationship that leads the wider society to treat both individuals in some respect as one". Thus, the family of patients with mental illness is all obliged to share some of the discredit of the stigmatized person to whom they are related (36). Accordingly, the stigma associated with mental disorders played a critical role in collaborative care and was required to be controlled through proper measures. Not only patients but also caregivers and HCPs suffered from the stigma linked to the treatment of and care for these patients. Some participants believed that stigma resulted from the lack of awareness about mental disorders and reported the necessity of educating society in this respect. Patrick W. Corrigan also conducted a meta-analysis in 2012 and introduced education as an important factor in decreasing the social stigma associated with patients with CMIs (37).

Stigma, in turn, led to hiding the disease on the part of the caregivers. Consistently, Pescosolido's (38) study pointed out that stigmatization of mental disorders led to discrimination for both patients and their caregivers. Thus, they emphasized the necessity to inform society to decrease this stigma (38). Another outcome of the existence of stigma was the lack of macro-level support for these patients, which was mentioned by some HCPs. They also emphasized the necessity to make attempts to attract policymakers' attention and prompt them to provide these patients with more support. Similarly, Kapungwe et al. (39) in a qualitative study conducted using 50 semi-structured interviews and 6 group discussions in Zambia, stigma, and discrimination associated with mental disorders were highly prevalent in the society, among families and HCPs, and at the government level. They conducted that it was necessary to change mental health policies and regulations, improve the patient's socioeconomic status, and create educational campaigns (39).

The disease's nature was yet another undeniable barrier mentioned by both caregivers and HCPs. Similarly, BaharCiftci et al. performed research in 2015 and indicated that patients suffering from mental disorders had problems in identifying their care needs, which revealed the need for periodical training programs for increasing the patients' self-care and improving their collaborative care (40).

The emergence of the COVID-19 pandemic was also another unexpected factor, which was considered an obstacle to family involvement in the care. The subcategories of this main category included limitations faced at hospitals due to the pandemic, performance of virtual rounds, lack of in-person training for families, and decline in the presence of families at hospitals and the associated problems. Evidence has also shown challenges in care processes due to the COVID-19 pandemic. Fabrizio Starace et al. also conducted a study in 2020 and indicated the importance of psychosocial interventions like home visits in open spaces (35). Consistently, Ann K. Shinn believed that although distance care approaches threatened health and behavioral care services for patients with CMIs, they were effective in eliminating the spread of the virus (41).

Organizational-structural factors as well as healthcare team dissociation in the care process were other barriers against family involvement in the care in the present investigation. Regarding organizational and structural factors, Giacco et al. (24) study informed that lack of cooperation on the part of caregivers might result from the lack of structured processes for identifying and contacting caregivers in the related organizations, which could convince the caregivers that other HCPs paid no attention to the value of their roles (24). The subcategories of this theme included communication problems between families and treatment teams, the system's teleological perspective, insufficient training as a barrier to collaborative care, and lack of HCP cooperation. Boyd (42), training HCPs in terms of communication techniques and teamwork was mentioned as the prerequisite for collaborative and interdisciplinary care (42). Giacco et al. (24) also maintained in their qualitative study that HCPs' problems in forging relationships with families originated from the lack of training for HCPs and insufficient monitoring while providing patients with interventions. Thus, they emphasized the necessity of education and proper supervision (24).

## Conclusion

Families are key members in the care of patients with chronic mental illnesses, but there are barriers in the way of this cooperation. Failure of the family to cooperate can cause repeated relapses and re-hospitalization of the patient. Families have to be helped for removing the family-related barriers of collaborative care, responsibility should be promoted toward the existing stigma in society, psychosocial interventions should be developed, the existing policies should be changed, organizational-structural factors should be eliminated, and plans should be made to train HCPs. Such training can improve caregivers' cooperation in patient care and, at the same time, lead to the development of more effective care programs with beneficial outcomes for patients with CMIs overall, the reported barriers emphasize the need for the development of structured approaches whose implementation is easy for HCPs, and will improve patient and family outcomes.

## Limitations and strong points

This research study was one of the first qualitative studies that assessed the viewpoints of caregivers, patients, and HCPs in terms of barriers and challenges of family involvement in the care of patients with CMIs referred to inpatient and outpatient treatment centers. This study pays special attention to key stakeholders involved in patient care that can be useful for managers and policymakers to use in care programs. However, this study had some limitations. Firstly, the participants were selected from a single geographical region (two megacities in XXX), which could affect the application of the results to other cultures. Another study limitation was the decline in the caregivers' presence and cooperation in healthcare centers due to the COVID-19 pandemic, which was somehow eliminated by prolonged engagement in the inpatient and outpatient settings. It is suggested that future studies be about family involvement in the care of other patients with chronic mental illnesses such as patients with obsessive-compulsive disorder and also in childhood such as autistic spectrum disorder.

## Data availability statement

The original contributions presented in the study are included in the article/Supplementary material, further inquiries can be directed to the corresponding author/s.

## Ethics statement

The studies involving human participants were reviewed and approved by Ethics approval of Isfahan University of Medical Sciences (IR.MUI.RESEARCH.REC.1399.502). The patients/participants provided their written informed consent to participate in this study.

## Author contributions

RD, MF-M, MSH, and EM-S were involved in study conception, design, and drafting of the manuscript. RD wrote the first draft of the manuscript. MF-M and MSH reviewed the first draft of the manuscript. EM-S was responsible for coordinating the study. All authors read and approved the final manuscript.

## Conflict of interest

The authors declare that the research was conducted in the absence of any commercial or financial relationships that could be construed as a potential conflict of interest. The reviewer SGH declared a shared affiliation with the authors RD and MF-M at the time of review.

## Publisher's note

All claims expressed in this article are solely those of the authors and do not necessarily represent those of their affiliated organizations, or those of the publisher, the editors and the reviewers. Any product that may be evaluated in this article, or claim that may be made by its manufacturer, is not guaranteed or endorsed by the publisher.

## References

[1] Wittchen HU Jacobi F Rehm J Gustavsson A Svensson M Jönsson B. The size and burden of mental disorders and other disorders of the brain in Europe 2010. Eur Neuropsychopharmacol. (2011) 21:655–679. 10.1016/j.euroneuro.2011.07.018 21896369

[2] Corrigan P Pickett S Kraus D Burks R Schmidt A. Community-based participatory research examining the health care needs of African Americans who are homeless with mental illness. J Health Care Poor Underserved. (2015) 26:119. 10.1353/hpu.2015.0018 25702732 PMC5388444

[3] Molu NG Ozkan B Icel S. Quality of life for chronic psychiatric illnesses and home care. Pakistan J |Med Sci. (2016) 32:511. 10.12669/pjms.322.8794 27182272 PMC4859055

[4] Charlson FJ Ferrari AJ Santomauro DF Diminic S Stockings E Scott JG. Global epidemiology and burden of schizophrenia: findings from the global burden of disease study 2016. Schizophr Bull. (2018) 44:1195–203. 10.1093/schbul/sby058 29762765 PMC6192504

[5] Liu Q He H Yang J Feng X Zhao F Lyu J. Changes in the global burden of depression from 1990 to 2017: findings from the global burden of disease study. J Psychiatr Res. (2020) 126:134–40. 10.1016/j.jpsychires.2019.08.002 31439359

[6] Ferrari AJ Stockings E Khoo JP Erskine HE Degenhardt L Vos T. The prevalence and burden of bipolar disorder: findings from the Global Burden of Disease Study 2013. Bipolar Disord. (2016) 18:440–50. 10.1111/bdi.12423 27566286

[7] Von Kardorff E Soltaninejad A Kamali M Eslami Shahrbabaki M. Family caregiver burden in mental illnesses: The case of affective disorders and schizophrenia–a qualitative exploratory study. Nord J Psychiatry. (2016) 70:248–54. 10.3109/08039488.2015.1084372 26524243

[8] Chevance A Ravaud P Tomlinson A Le Berre C Teufer B Touboul S. Identifying outcomes for depression that matter to patients, informal caregivers, and health-care professionals: qualitative content analysis of a large international online survey. Lancet Psychiatry. (2020) 7:692–702. 10.1016/S2215-0366(20)30191-7 32711710

[9] Fisher A Sharpe L Anderson J Manicavasagar V Juraskova I. Development and pilot of a decision-aid for patients with bipolar II disorder and their families making decisions about treatment options to prevent relapse. PLoS ONE. (2018) 13:e0200490. 10.1371/journal.pone.0200490 29990368 PMC6039033

[10] Kumar D Ashwini K Hegde S Prasanna L Joseph B Bose A. Caregiver assisted home-based cognitive remediation for individuals diagnosed with schizophrenia: A pilot study. Asian J Psychiatr. (2019) 42:87–93. 10.1016/j.ajp.2019.03.010 30981943 PMC7613146

[11] WHO(2014). Integrating the Response to Mental Disorders and Other Chronic Diseases in Health Care Systems. Geneva, Switzerland: World Health Organization.

[12] Vigo D Thornicroft G Atun R. Estimating the true global burden of mental illness. Lancet Psychiatry. (2016) 3:171–8. 10.1016/S2215-0366(15)00505-2 26851330

[13] Wong OL Wan ESF Ng MLT. Family-centered care in adults' mental health: Challenges in clinical social work practice. Soc Work Ment Health. (2016) 14:445–64. 10.1080/15332985.2015.1038413

[14] NICE(2012). Service User Experience in Adult Mental Health: NICE Guidance on Improving the Experience of Care for People Using Adult NHS Mental Health Services. London: National Collaborating Centre for Mental Health.

[15] Tantirangsee N Assanangkornchai S Marsden J. Effects of a brief intervention for substance use on tobacco smoking and family relationship functioning in schizophrenia and related psychoses: A randomised controlled trial. J Subst Abuse Treat. (2015) 51:30–7. 10.1016/j.jsat.2014.10.011 25468004

[16] Gray B Robinson C Seddon D Roberts A. ‘Confidentiality smokescreens' and carers for people with mental health problems: the perspectives of professionals. Health Soc Care Commun. (2008) 16:378–87. 10.1111/j.1365-2524.2007.00748.x 18194286

[17] Askey R Holmshaw J Gamble C Gray R. What do carers of people with psychosis need from mental health services? Exploring the views of carers, service users and professionals. J Family Therapy. (2009) 31:310–31. 10.1111/j.1467-6427.2009.00470.x

[18] Semrau M Lempp H Keynejad R Evans-Lacko S Mugisha J Raja S. Service user and caregiver involvement in mental health system strengthening in low-and middle-income countries: systematic review. BMC Health Serv Res. (2016) 16:1–18. 10.1186/s12913-016-1323-8 26931580 PMC4774091

[19] Wilkinson C McAndrew S. ‘I'm not an outsider, I'm his mother!'A phenomenological enquiry into carer experiences of exclusion from acute psychiatric settings. Int J Ment Health Nurs. (2008) 17:392–401. 10.1111/j.1447-0349.2008.00574.x 19128286

[20] Jankovic J Yeeles K Katsakou C Amos T Morriss R Rose D. Family caregivers' experiences of involuntary psychiatric hospital admissions of their relatives–a qualitative study. PloS One. (2011) 6:e.25425. 10.1371/journal.pone.0025425 22022393 PMC3192057

[21] Nurjannah I Mills J Usher K Park T. Discharge planning in mental health care: an integrative review of the literature. J Clin Nurs. (2014) 23:1175–85. 10.1111/jocn.12297 23844598

[22] Hsiao CY Tsai YF. Factors associated with the perception of family nursing practice among mental health nurses in Taiwan. J Family Nurs. (2015) 21:508–28. 10.1177/1074840715606543 26410853

[23] Rose LE Mallinson RK Walton-Moss B. Barriers to family care in psychiatric settings. J Nurs Scholarship. (2004) 36:39–47. 10.1111/j.1547-5069.2004.04009.x 15098417

[24] Giacco D Dirik A Kaselionyte J Priebe S. How to make carer involvement in mental health inpatient units happen: a focus group study with patients, carers and clinicians. BMC Psychiatry. (2017) 17:1–13. 10.1186/s12888-017-1259-5 28320376 PMC5359804

[25] Graneheim UH Lundman B. Qualitative content analysis in nursing research: concepts, procedures and measures to achieve trustworthiness. Nurse Educ Today. (2004) 24:105–12. 10.1016/j.nedt.2003.10.001 14769454

[26] Polit DF Beck CT. Nursing Research: Principles and Methods. Philadelphia, PA: Lippincott Williams and Wilkins. 2004.

[27] LoGerfo JP. Explorations in quality assessment and monitoring. Volume I: the definitions of quality and approaches to its assessment. Med Care. (1981) 19:1066–7. 10.1097/00005650-198110000-00010

[28] Vimala S. W. (2011). Practical Guide to Mental Health Nursing. New Delhi, India: Jaypee Brothers Publishers.

[29] Cohen AN Glynn SM Hamilton AB Young AS. Implementation of a family intervention for individuals with schizophrenia. J Gen Intern Med. (2010) 25:32–7. 10.1007/s11606-009-1136-0 20077149 PMC2806956

[30] Clifton A Hemingway A Felton SA Stacy G. Fundamental of Mental Health Nursing an Essential Guide for Nursing and Health Care Students. Hoboken, NJ: Wiley, Blackwell (2018).

[31] Townsend MC. Essentials of Psychiatric Mental Health Nursing: Concepts of Care in Evidence-Based Practice. Philadelphia, PA: FA Davis (2013).

[32] Feldman DB Crandall CS. Dimensions of mental illness stigma: what about mental illness causes social rejection? J Soc Clin Psychol. (2007) 26:137–54. 10.1521/jscp.2007.26.2.137

[33] Pharoah F Mari JJ Rathbone J Wong W. Family intervention for schizophrenia. Cochrane Database Syst Rev. (2010) CD000088. 10.1002/14651858.CD000088.pub3 17054127

[34] Girón M Nova-Fernández F Mañá-Alvarenga S Nolasco A Molina-Habas A Fernández-Yañez A. How does family intervention improve the outcome of people with schizophrenia? Soc Psychiatry Psychiatr Epidemiol. (2015) 50:379–87. 10.1007/s00127-014-0942-9 25087012

[35] Starace F Ferrara M. COVID-19 disease emergency operational instructions for mental health departments issued by the italian society of epidemiological psychiatry. Epidemiol Psychiatr Sci. (2020) 29:e116. 10.1017/S2045796020000372 32228737 PMC7163186

[36] Goffman E. Stigma: Notes on the Management of Spoiled Identity. New York, NY: Simon & Schuster (2009).

[37] Corrigan PW Morris SB Michaels PJ Rafacz JD Rüsch N. Challenging the public stigma of mental illness: a meta-analysis of outcome studies. Psychiatric Serv. (2012) 63:963–73. 10.1176/appi.ps.201100529 23032675

[38] Pescosolido BA. The public stigma of mental illness: What do we think; what do we know; what can we prove? J Health Soc Behav. (2013) 54:1–21. 10.1177/0022146512471197 23325423 PMC4437625

[39] Kapungwe A Cooper S Mwanza J Mwape L Sikwese A Kakuma R. Mental illness-stigma and discrimination in Zambia. Afr J Psychiatry. (2010) 13:192–203. 20957318

[40] Çiftçi B Yildirim N Altun ÖS Avşar G. What level of self-care agency in mental illness? The factors affecting self-care agency and self-care agency in patients with mental illness. Arch Psychiatric Nurs. (2015) 29:372–6. 10.1016/j.apnu.2015.06.007 26577549

[41] Shinn AK Viron M. Perspectives on the COVID-19 pandemic and individuals with serious mental illness. J Clin Psychiatry. (2020) 81:0–0. 10.4088/JCP.20com13412 32369691

[42] Boyd MA. Essential of Psychiatric Nursing. Alphen aan den Rijn, Netherlands: Wolter kluwer (2017).

