# Supplementary material for: Barriers to family involvement in the care of patients with chronic mental illnesses: A qualitative study
Source: Front Psychiatry. 2022 Oct 19;13:995863. doi: 10.3389/fpsyt.2022.995863 (PMC9627781; doi:10.3389/fpsyt.2022.995863)
Supplement: Supplementary file 1 [file Table_1.DOCX]

**Consolidated criteria for reporting qualitative studies (COREQ): 32-item checklist**

**Barriers to** **family involvement in the care of patients with chronic mental illnesses: A qualitative study**

| **Domain 1: Research team and reflexivity** | |
| --- | --- |
| **Personal Characteristics** | |
| 1. Interviewer/facilitator | Which author/s conducted the interview or focus group? First, second and third authors |
| 2. Credentials | What were the researcher’s credentials? PhD(first author) candidate, PhD(second and third authors) and Psychiatrist(Fourth author) |
| 3. Occupation | What was their occupation at the time of the study? First author is nursing PhD student and others are university professor. |
| 4. Gender | Was the researcher male or female? First author is female and others are male. |
| 5. Experience and training | What experience or training did the researcher have? The second till fourth authors are professor at the University of Medical Sciences and has many years of experience in quantitative and qualitative research, and first author is PhD student who have already been researchers. |
| **Relationship with participants** | |
| 6. Relationship established | Was a relationship established prior to study commencement? yes |
| 7. Participant knowledge of the interviewer | What did the participants know about the researcher? The goals and reasons for the research were explained to them |
| 8. Interviewer characteristics | What characteristics were reported about the interviewer/facilitator? Work experience, interest in entering the study |
| **Domain 2: study design** | |
| **Theoretical framework** | |
| 9. Methodological orientation and Theory | What methodological orientation was stated to underpin the study? qualitative content analysis |
| **Participant selection** | |
| 10. Sampling | How were participants selected? purposive |
| 11. Method of approach | How were participants approached? face-to-face |
| 12. Sample size | How many participants were in the study? 34 participants |
| 13. Non-participation Setting | How many people refused to participate or dropped out? Reasons? A number of participants were asked to cooperate in the interview, but some expressed their unwillingness due to their busy schedule |
| 14. Setting of data collection | Where was the data collected? hospital |
| 15. Presence of non-participants | Was anyone else present besides the participants and researchers? No |
| 16. Description of sample Data collection | What are the important characteristics of the sample? semi-structured interviews were conducted with 34 participants including patients, informal caregivers, and health care providers from 30 December 2020 to 25 August 2021 |
| 17. Interview guide | Were questions, prompts, guides provided by the authors? Yes  Was it pilot tested? No |
| 18. Repeat interviews | Were repeat interviews carried out? If yes, how many? No |
| 19. Audio/visual recording | Did the research use audio or visual recording to collect the data? Audio recording was done |
| 20. Field notes | Were field notes made during and/or after the interview or focus group? yes |
| 21. Duration | What was the duration of the interviews or focus group? 30-70 minutes |
| 22. Data saturation | Was data saturation discussed? yes |
| 23. Transcripts returned | Were transcripts returned to participants for comment and/or correction? yes |
| **Domain 3: analysis and findings** | |
| **Data analysis** | |
| 24. Number of data coders | How many data coders coded the data? 3 |
| 25. Description of the coding tree | Did authors provide a description of the coding tree? yes |
| 26. Derivation of themes | Were themes identified in advance or derived from the data? derived from the data |
| 27. Software | What software, if applicable, was used to manage the data? MAXQDA software (2018) was used for coding and organizing the data. |
| 28. Participant checking Reporting | Did participants provide feedback on the findings? Yes member check was done |
| 29. Quotations presented | Were participant quotations presented to illustrate the themes / findings? Was each quotation identified? Yes each narratives has participant number |
| 30. Data and findings consistent | Was there consistency between the data presented and the findings? Yes data presentation and findings are consistent |
| 31. Clarity of major themes | Were major themes clearly presented in the findings? Yes clearly |
| 32. Clarity of minor themes | Is there a description of diverse cases or discussion of minor themes? yes |
